# Exploring disability from the perspective of adults living with HIV/AIDS: Development of a conceptual framework

**DOI:** 10.1186/1477-7525-6-76

**Published:** 2008-10-04

**Authors:** Kelly K O'Brien, Ahmed M Bayoumi, Carol Strike, Nancy L Young, Aileen M Davis

**Affiliations:** 1 Department of Health Policy, Management and Evaluation, University of Toronto, Toronto, Ontario, Canada; 2 Centre for Research on Inner City Health, St. Michael's Hospital, 30 Bond Street, Toronto, Ontario, M5B 1X8, Canada; 3 Department of Psychiatry, University of Toronto, Toronto, Ontario, Canada; 4 Centre for Addiction and Mental Health, 33 Russell St., 3rd Floor Tower, Toronto, Ontario, M5S 2S1, Canada; 5 School of Rural and Northern Health, Laurentian University, 935 Ramsey Lake Road, Sudbury, Ontario, P3E 2C6, Canada; 6 Division of Health Care and Outcomes Research and Arthritis and Community Research and Evaluation Unit, Toronto Western Research Institute, 399 Bathurst Street - MP11-322, Toronto, Ontario, M5T 2S8, Canada

## Abstract

**Background:**

Since the advent of combination antiretroviral therapy, in developed countries HIV increasingly is perceived as a long-term illness. Individuals may experience health-related consequences of HIV and its associated treatments, a concept that may be termed disability. To date, a comprehensive framework for understanding the health-related consequences experienced by people living with HIV has not been developed. The purpose of this research was to develop a conceptual framework of disability from the perspective of adults living with HIV.

**Methods:**

We conducted four focus groups and 15 face-to-face interviews with 38 adults living with HIV. We asked participants to describe their health-related challenges, their physical, social and psychological areas of life affected, and impact on their overall health. We analyzed data using grounded theory techniques. We also conducted two validity check focus groups with seven returning participants.

**Results:**

Disability was conceptualized by participants as multi-dimensional and episodic characterized by unpredictable periods of wellness and illness. The *Episodic Disability Framework *consisted of three main components: a) dimensions of disability that included symptoms and impairments, difficulties carrying out day-to-day activities, challenges to social inclusion, and uncertainty that may fluctuate on a daily basis and over the course of living with HIV, b) contextual factors that included extrinsic factors (social support and stigma) and intrinsic factors (living strategies and personal attributes) that may exacerbate or alleviate disability, and c) triggers that initiate momentous or major episodes of disability such as receiving an HIV diagnosis, starting or changing medications, experiencing a serious illness, and suffering a loss of others.

**Conclusion:**

The *Episodic Disability Framework *considers the variable nature of disability, acknowledges uncertainty as a key component, describes contextual factors that influence experiences of disability, and considers life events that may initiate a major or momentous episode. This framework presents a new way to conceptualize disability based on the experience of living with HIV.

## Background

Since the introduction of combination antiretroviral therapy, in developed countries HIV increasingly is perceived as a long-term illness in which individuals experience complications of both the disease and its associated treatments [1]. Characterizing such challenges, which may be termed disability, is necessary for a comprehensive evaluation of the experience of living with HIV and for improving associated care, treatment and support.

Existing definitions of disability lack clarity and are often contextual. For example, in the employment context, disability may be defined as a person's ability to work, while in the health care context, disability may be defined as a person's physical ability to carry out a life-related task. The lack of a unifying framework for considering disability can therefore lead to confusion about definitions, poor communication, and fragmented service delivery [2,3].

To date, a comprehensive framework for understanding the health-related consequences experienced by people living with HIV has not been developed. Four frameworks conceptualize disability as a component of a larger set of limitations at the individual [4,5] or societal level [6-8]. A recent version of the World Health Organization framework defines disability broadly, encompassing 'impairments, activity limitations and participation restrictions' (p.10) that span body, individual and societal perspectives [9]. None of the five frameworks was developed specifically for HIV and all were established prior to the advent of combination antiretroviral therapy. Furthermore, it is unclear whether a generic illness perspective can accurately capture the complexity of an illness that is evolving as medications are changing and life expectancy increasing. Hence, the details of the day-to-day health-related consequences of HIV – a concept that may be provisionally termed as *disability *– and their significance from the perspective of people living with HIV are unknown. The purpose of this research was to develop a conceptual framework of disability from the perspective of adults living with HIV.

## Methods

While we approached this research with a broad conceptualization of disability developed from existing disablement frameworks, our goal was to construct a framework built from the experiences of living with HIV. Accordingly, we had an initial definition of disability – *the day-to-day challenges that persons living with HIV experience as a result of the disease, its associated conditions and treatments *– but avoided using this term in our consultations with participants. Throughout the research process, we aimed to remain open to more relevant terms and sought definitions of disability that emerged from the perspective of adults living with HIV.

We recruited HIV-positive men and women to participate in focus groups and interviews to discuss the concept of disability. Participants, who had to be over 18 and self-identify as having experienced at least one health-related consequence attributed to their illness, were recruited from an academic hospital, a primary care clinic, and a number of local AIDS Service Organizations. Written informed consent was received from all participants. This research was guided by a Community Advisory Committee and approved by the St. Michael's Hospital and University of Toronto Research Ethics Boards.

### Focus Group and Interview Procedures

This research consisted of three phases of inquiry: pilot focus groups, semi-structured interviews, and validity check focus groups. The goal of the pilot focus groups was to guide the sampling strategy and refine the interview guide for the interview phase of the research; these data also contributed to the development of the conceptual framework. After analyzing the pilot focus group transcripts, we conducted face-to-face interviews with new participants. The goal of the interviews was to gather data to establish the conceptual framework of disability. The interview guide consisted of open-ended questions and participants were asked to describe their limitations, restrictions and health-related challenges living with HIV, physical, social and psychological areas of their life affected, and how these challenges affected their overall health. Over the course of the interviews and the constant comparative analysis, we revised the interview guide to include probing questions asking participants about living with HIV, the associated uncertainty, and strategies participants used to deal with their daily health-related challenges. We avoided using the term 'disability' until the end of the interviews when we asked participants what the term meant to them as it related to living with HIV.

Finally, all interview participants were invited to participate in a validity check focus group discussion. The goal of the validity check focus groups was to enhance, refine, and refute the emerging framework [10]. Participants were asked to comment on the content, terminology and interrelationships within the framework to see if it adequately represented their experiences living with HIV.

Interviews and focus group discussions were conducted at three community-based organizations in Toronto. All discussions were audio taped and field notes taken throughout for later verbatim transcription and analysis. Data management was facilitated using N6 computer software. All participants completed a demographic questionnaire and the HIV Symptom Index [11].

#### Analysis

The paradigm for understanding disability in this study was an interpretive qualitative one [12]. The analytical technique was grounded theory, based on the principles of Strauss and Corbin [13]. We used a systematic set of procedures which included: breaking transcript data down line-by-line into concepts termed meaning units (open coding); grouping similar meaning units into categories and identifying their properties and dimensions; comparing categories with other categories; identifying relationships between categories (axial coding); and integrating these categories with the construct of disability to develop the theory (selective coding) [13]. We used a constant comparative method of analysis whereby data collection and analysis occurred simultaneously.

We used a deductive approach to analyze the validity check focus group data. We examined the data for ways to enhance and refine the content, terminology and interrelationships of the preliminary framework and to determine whether it adequately represented the experiences of adults living with HIV [13].

Theoretical saturation, constant comparative analysis and validity checks were used to enhance rigor [14]. Sampling until theoretical saturation ensured data collection continued until no 'new' relevant data emerged. Constant comparative analysis ensured discussion guides continually evolved so that questions adequately built and strengthened the theory. Validity checking with the final focus groups involved sharing preliminary results with the participants, and enabling them to confirm, refute or, enhance the hypothetical framework from the interviews. Validity checking also occurred through the independent assignment of meaning units and categories with three transcripts by three authors and a colleague with expertise in qualitative research. Interim data and analytical interpretations were formally reviewed among authors and a Community Advisory Committee six times over the course of 14 months.

## Results

Thirty-eight participants took part in one of four focus groups or 15 face-to-face interviews (Table 1). Three men and four women interview participants returned to participate in a validity check focus group discussion.

**Table 1:** Characteristics of Focus Group and Interview Participants (n = 38)

**Characteristic**	**Number (%)**
*Gender*	
Male	21 (55%)
Female	16 (42%)
Transgendered	1 (3%)

Age	41 years old (range: 27–58 years)

Identified with particular ethnic group	23 (60%)**

Nadir CD4 count < 200 cells/mm3	19 (50%)

Diagnosed Prior to 1996	17 (45%)

Experienced an HIV-related illness	11 (73%)*†

Currently Taking HIV Medications	25 (66%)

Currently Working	6 (40%)* (3 full-time, 3 part-time)

*Self Rated Health Status*	
Poor	0 (0%)
Fair	2 (5%)
Good	16 (42%)
Very Good	15 (39%)
Excellent	5 (14%)

*HIV Symptom Index*	
Median Number of Symptoms Present	15/20 (IQR: 8–18)
Median Number of Bothersome Symptoms	13/20 (IQR: 8–18)^

*denominator of 15 interview participants only

**13 identified themselves as African/African Caribbean/Black African/Black; 2 Jewish; 1 West Indian; 1 Latin; 1 Italian Canadian; 1 Irish Canadian; 1 French Italian; 1 English British; 1 White Caucasian; 1 not specified.

†most common HIV-related illness included pneumonia/lung infection/PCP pneumonia (n = 6 participants).

^Most bothersome symptoms included fatigue or loss of energy, feeling sad, down or depressed, and feeling nervous or anxious.

IQR = interquartile range

### Conceptualization of Disability: Multi-dimensional and Episodic

Participants' conceptualizations of disability emerged as multi-dimensional and episodic in nature. These two attributes of disability served as the foundation for the conceptual framework.

Disability spanned physical, mental, emotional, and social life domains. It included both *"visible" *and *"invisible" *components representing physical and emotional health challenges respectively:

"For me, disabled is not being able to keep up, not being able to fully function, and feeling the guilt, and feeling the sadness and the emptiness, the loss. That's disability – just feeling exhausted and worn out" (INT-1)

Sometimes, disability was referred to within a mental health context. Despite feeling physically healthy, participants described how an HIV diagnosis represented a continual burden that reminded them of how their lives were forever affected by the disease:

"It's just a disability in your mind cause it plays at your mind... because your brain constantly has to deal with new aspects, new feelings, and new sensations... Your brain is always working, 24 hours..." (INT-7)

Other conceptualizations of disability emerged within a social context. Limitations related to finances and the inability to access needed services such as housing, medications, or insurance, all reduced an individual's ability to participate in society:

"I think what holds a lot of people back is the cost of the drugs, the medications, that's the freaking disability! The way you can't get life insurance...you can't have the same things. You can't buy a house because nobody will give you a mortgage... That's a disability – the system." (INT-5)

Episodic disability was characterized by unpredictable periods of wellness and illness. Episodes of disability were described as health-related setbacks that manifested from HIV disease or its associated treatments. Disability was not an absolute state, but rather a fluctuating range of types of health-related challenges. Participants articulated that *"nothing's constant with HIV" *and described HIV as a *"roller coaster" *and an *"up and down" *experience.

Episodes occurred at irregular intervals on a daily basis and over the entire course of living with HIV. Major episodes represented large or momentous health variations over the health trajectory that sometimes included a hospitalization, whereas minor episodes were described as "*good days and bad days*" that represented day-to-day fluctuations in health. For many, episodes were not considered cumulative; rather participants described how they often regained their overall health, or even considered themselves healthier after overcoming an episode.

### Disability Terminology

One goal of this study was to ascertain whether the term disability accurately represented the health-related challenges experienced living with HIV. Participants perceived disability as a term which suggested permanency, in contrast to their experiences of episodic illness. However participants accepted this label in certain situations to access needed services:

*"The word disability is not an offensive word to me. I just find it inaccurate. Disability implies a constant unchanging state whereas HIV related challenges are more fluid. I would describe HIV as a chronic fluctuating illness*...*We're responding to a system that says you're either disabled or you're not disabled. If you're not disabled, you don't get certain benefits. You don't get certain acknowledgement." (INT-10)*

Rather than a "*permanent*", "*all or nothing*", or "*concrete*" state of health, participants described how disability "*fluctuates*," is *"inconsistent*" reflecting its episodic nature that consists of a range of levels and types of health-related challenges. Alternatively, participants referred to themselves as "*part-time disabled, once in a while." *Hence, there was a need to respond to the inaccuracy of the term disability used alone, as well as acknowledge the significant challenges experienced by adults living with HIV. Consequently, the term *episodic disability *emerged as a more accurate term to conceptualize the variable health-related consequences experienced by adults living with HIV.

### Episodic Disability Framework

**Figure 1:**
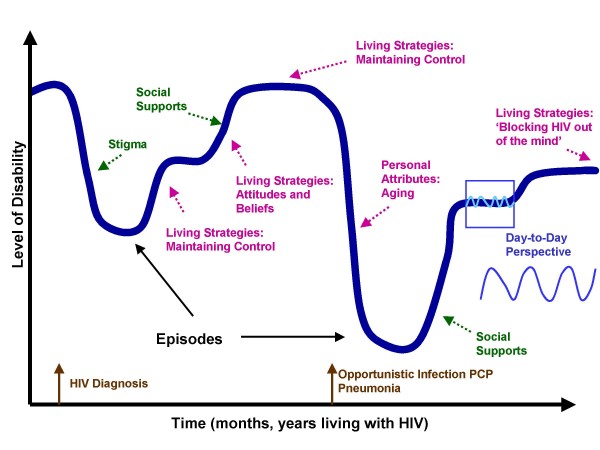
**Episodic Disability Framework: **An example of a person's disability experience illustrating the episodic nature of disability that occurs on a daily basis and over the entire course of living with HIV. Episodes of disability may be triggered by life events (brown), and exacerbated or alleviated by extrinsic contextual factors (green) and intrinsic contextual factors (pink).

We developed the *Episodic Disability Framework *to broadly conceptualize the way in which the multi-dimensional health-related challenges are experienced by adults living with HIV. This framework describes disability across a trajectory of a range of types and levels of disablement (Figure 1). The trajectory includes the day-to-day health fluctuations, or 'good days' and 'bad days', super-imposed along the larger course of disablement that may occur over months or years of living with HIV. Three main components comprise the *Episodic Disability Framework*: a) dimensions of *episodic disability*, b) factors that describe the context in which disability is experienced, and c) triggers that may initiate a major or momentous episode of disability.

#### A. Dimensions of Episodic Disability

**Figure 2:**
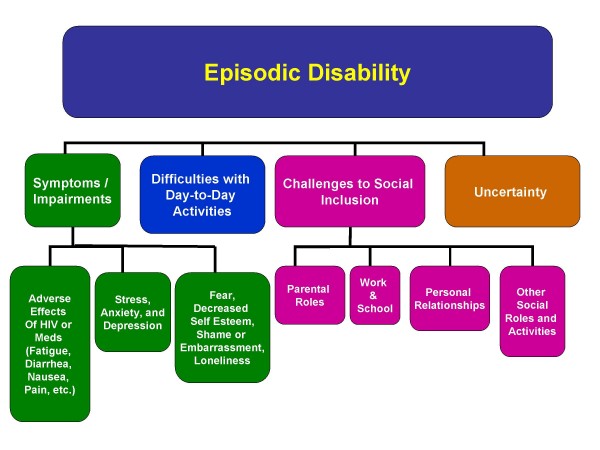
**Dimensions of Episodic Disability: **Four dimensions of *episodic disability *and their sub-components that may be experienced by adults living with HIV.

*Episodic disability *was defined as the consequences of HIV and its treatments including symptoms and impairments, difficulties carrying out day-to-day activities, challenges to social inclusion, and uncertainty that may fluctuate on a daily basis and over the entire course of living with HIV (Figure 2). We describe each of these four dimensions in Additional File 1.

**Figure 3:**
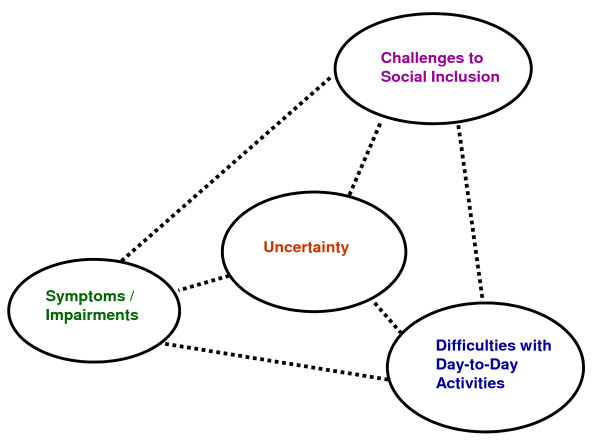
**Potential Linkages Between Dimensions of Disability: **Symptoms and impairments, difficulties with day-to-day activities, challenges to social inclusion and uncertainty appeared to be linked; meaning a change in one dimension was associated with a change in the other.

All four disability dimensions appeared to be linked; meaning the experience in one dimension was associated with the experience of another (Figure 3). For example, an exacerbation of a symptom or impairment (such as fatigue), was sometimes associated with a person's social inclusion (such as his/her ability to work). Alternatively, a reduction in a symptom or impairment (such as fatigue) was sometimes associated with an increased ability to carry out day-to-day activities (such as obtaining groceries). Exacerbation of uncertainty was sometimes associated with increased levels of stress, anxiety and depression as participants worried about the future.

These dimensions provide a description of disability, but do not contribute to the understanding of *how *disability is experienced by adults living with HIV. This is addressed in the two sections below.

#### B. Contextual Factors of Disability

**Figure 4:**
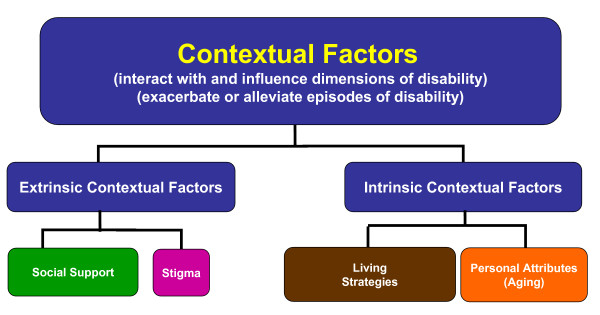
**Contextual Factors of Disability: **Factors that describe the context in which disability is experienced. Extrinsic and intrinsic contextual factors could exacerbate or alleviate episodes of disability for adults living with HIV.

Throughout the accounts, participants described their health-related challenges in relation to the features that altered their experiences, termed contextual factors. Participants spoke of two groups of extrinsic and intrinsic contextual factors that could exacerbate or alleviate their disability (Figure 4).

Extrinsic factors included social support and stigma. Social support included practical and emotional support provided by, or received from friends, family, partners, pets and the community; support from accessing health care services and personnel; and program and policy support. Stigma resulted from discrimination from friends, family, work colleagues, employers and health care providers due to a participants' HIV status, their sexual orientation, ethnocultural background, employment status, and/or gender and exacerbated disability.

Intrinsic factors included living strategies and personal attributes. Living strategies were behaviours, attitudes and beliefs participants adopted in order to deal with HIV and its resulting disablement. Strategies included seeking interactions with others, maintaining a sense of control over life and the illness, 'blocking HIV out of the mind', and adopting attitudes and beliefs to help manage living with HIV. Personal attributes, referred to non-modifiable characteristics such as age and co-morbid illnesses inherent to an individual. The contextual factors could be static (either present or absent) (e.g. social policy), progressive (e.g. aging) or fluctuate over time (e.g. levels of support). Based on the nature and extent of their presence or absence, these contextual factors influenced the episodic nature of the dimensions of disability.

#### C. Triggers of Disability

Participants described at length the events that initiated major or momentous episodes of disability, which constituted the final component of the framework. Four examples of disability triggers included: receiving an HIV diagnosis; starting or changing antiretroviral medications; experiencing a serious illness; and suffering a loss of others (Table 2).

**Table 2:** Examples of Triggers of Disability

**Receiving an HIV Diagnosis**
Receiving news of an HIV diagnosis marked a severe episode of disability. Participants reflected how this one-time episode was a life-changing event that initiated life with HIV and its uncertainty.
*"From the point of diagnosis you deal with depression... coming to grips with realizing that the virus is attacking your body...And how that changes your life completely." (INT-11)*

**Initiating or Changing Antiretroviral Medications**

Participants described the complexity of weighing the physiological benefits of medications with the potential adverse effects. Some were fearful of how they might react to antiretrovirals, specifically the physical effects that could result, identifying them as HIV-positive, and making them vulnerable to stigma and discrimination:
*"I've gone through the whole process of choosing my medications...And there's still a lot of uncertainties...Is this going to cause body side effects, which I'm terrified about because that will change my life drastically. Because I think the only reason that I've felt in a sense somewhat normal – I wouldn't say whole normal at all – is that I don't look any different..." (INT-11)*

**Experiencing a 'Serious Illness'**

These included illnesses related to HIV or co-morbidities participants were living with prior to being diagnosed with HIV. Examples included osteoarthritis, osteoporosis, Hepatitis-C co-infection, lipodystrophy, diabetes, stroke, myocardial infection, and pneumonia.

**Suffering a Loss of Others**

Losing a family member, friend, or partner (regardless of whether attributed to HIV) sparked uncertainty as participants began worrying about their own health and survival.
*"it actually does cause me a bit of a dip when I notice a neighbor getting sick. I live in a building of all people with HIV and in the past few years 4 or 5 people died... and I went for a dip each death, even if I didn't know people...what happens to them matters and it actually affects me... I feel like my immune system is touched when that happens, I just get so down...that weighs on me." (INT-10-VCFG-1)*

## Discussion

Findings from this thesis include a new framework for understanding disability in the context of HIV. Results indicate that disability is both multi-dimensional and episodic in nature. Accordingly, we encourage the use of the term *episodic disability *to classify and conceptualize the health-related consequences of living with HIV. While the chronic illness literature has recognized that more minor health fluctuations (good and bad days) are superimposed upon major illness trajectories, such considerations are infrequent in disablement frameworks [15,16]. With the exception of the term 'process' used by Verbrugge and Jette [8] and Fougeyrollas and colleagues [5], to acknowledge a course of disablement, most frameworks conceptualize disability at a single point in time [4,6,7,9].

Episodes of disablement were central to the illness experience. Their meanings were determined by whether participants classified episodes as major or minor and how they impacted their health. Major episodes were associated with triggers that marked life events such as a serious illness whereas others were redefined as common manageable occurrences or minimal intrusions on everyday life after living with HIV for many years. Despite this redefinition, the day-to-day health-related consequences of HIV often posed considerable disablement. Hence, the variability in episode length and severity, and the way in which daily fluctuations may be superimposed on the more major or momentous events are an important feature of this framework.

### Dimensions of Disability

We used negative labels for the dimensions of disability based on the language preferred by participants. This approach is consistent with previous disablement frameworks [4-8] but in contrast to the *Handicap Creation Process *and *International Classification of Functioning, Disability and Health *(ICF) which adopted positive terminology to reduce the stigma associated with disability [5,9]. Participants in this study felt that acknowledging health-related challenges as negative was important to ensure that health systems, programs and policies respond adequately to the needs of people living with HIV [17].

Uncertainty is a new dimension of disability arising from this study. The three remaining dimensions are represented within existing disablement frameworks. Symptoms and impairments are analogous to disablement at the level of the body part, structure or function [4-9]. Difficulties carrying out day-to-day activities are analogous to functional limitations [6-8], disability [4,5] and activity limitations [9]. Finally, challenges to social inclusion are analogous to disability [6-8], handicap [4,5] and participation restrictions [9].

Uncertainty is a well recognized source of emotional distress, fear, anxiety, and depression for people living with HIV [18-24]. With medical advances, individuals living with HIV often faced new uncertainties as they struggled to come to terms with planning for life rather than imminent death. Many had to renegotiate their life priorities and re-construct their identities as a person living with a long-term illness. Hence, the uncertainty of living with a chronic illness may be considered as challenging as the knowledge of impending death [25].

Brashers and colleagues [26] define uncertainty as an "individual's inability to ascribe meaning to illness when outcomes are unpredictable and when the disease and its treatments and symptoms of care are ambiguous, highly complex and lacking information." *Mishel's Uncertainty in Illness Trajectory *[27], conceptualizes uncertainty as the social, emotional and interpersonal unknowns associated with diagnosis, disease progression, and long term prognosis. These sources of uncertainty were similarly seen in this research, and while we did not set out to determine whether individuals could assign meaning to their illness like Brashers and colleagues [26], we found that participants were able to articulate the impact uncertainty had on their overall health.

### Contextual Factors and Triggers of Disability

Disablement frameworks were determined unable to fully conceptualize the complex risk factors and intermediary steps in the process of disablement. The *Nagi Scheme *[6,7], and the *International Classification of Impairments, Disabilities and Handicaps (ICIDH) *[4] do not elaborate on the context in which disablement may be experienced. In contrast, the *Handicap Creation Process *[5] highlights the individual identity and social context in which handicap is experienced, specifically environmental factors that can either be an 'obstacle' or 'support' to an individual's level of function [5]. Similarly, the *Disablement Process *describes intra- and extra-individual factors that exacerbate/alleviate, or accelerate/decelerate the disablement process [8], and the *ICF *describes personal and environmental factors, that interact with and affect a person's functioning and health [9]. However, these three frameworks do not describe in detail how the factors influence dimensions of disablement. The *Episodic Disability Framework *describes the intrinsic and extrinsic contextual factors that influence the nature and severity of disability episodes. Understanding how these factors may be modified might present strategies for people living with HIV and their health care providers to prevent or reduce disablement.

Existing disablement frameworks do not account for life events associated with a phase of transition from wellness to illness and vice versa. For example, we found that receiving an HIV diagnosis was characterized as an event that forever altered a person's life that, for some, was associated with anxiety, depression, devastation and hopelessness [20,28,29]. Over time, individuals may adjust to their disease, adopt a greater appreciation for life, pay closer attention to their health, and re-establish relationships with family [30]. Individuals may even perceive their health as improved since becoming infected with HIV [31].

### Linkages Among Dimensions of Disability

Interpretations of the data suggest that the four dimensions of disability are linked. Given exploration of relationships within the framework was not a purpose of this research; we did not specifically probe participants about potential associations. Hence we were unable to determine directional relationships that may exist among specific dimensions in the *Episodic Disability Framework *(Figure 3). While symptoms and impairments, difficulties with day-to-day activities and challenges to social inclusion appeared bidirectionally linked (similar to components in the *ICF*), the relationships of these dimensions with uncertainty were less clear.

### Implications for Clinicians, Patients, Researchers and Policy

The *Episodic Disability Framework *possesses practical implications for clinicians, patients, researchers and policy makers. Clinicians can use this framework to structure their thinking about the dimensions of HIV-related disability, particularly those dimensions that may not have been previously well recognized such as living with uncertainty. Adults living with HIV may use the framework to better articulate their experiences to care providers. The framework also offers living strategies that individuals may use to mitigate disability episodes. Researchers may use the *Episodic Disability Framework *to guide the development of future measurement tools, particularly noting the importance of capturing the context in which disability is experienced and investigating how to capture the episodic nature of disability over time. Policy makers might consider how recognizing the episodic nature of disability could lead to more flexible income and labour force policies and programs.

This study has some limitations. We focused exclusively on English speaking men and women, primarily living in central Toronto, potentially limiting the perspectives of disability if these individuals are not representative of others living with HIV. Participation bias also may exist if those who volunteered conceptualized disability differently from those who chose not to participate. For example, aboriginal Canadians, youth and persons from rural geographic regions were not represented in this sample; exploring the generalizability of the framework for these groups and other populations is an important topic for future research. In addition, we did not specifically analyze how the severity of HIV illness might relate to disability, although it is important to note that traditional measures of severity (CD4 count, past AIDS illness) might have less significance with current antiretroviral therapy options.

The linkages between dimensions of *episodic disability *are merely descriptive in nature, and while the participants implied associations between these dimensions, we do not have data to suggest directional nor causal relationships. Empirical testing is needed to explore these linkages, determine whether they are bidirectional or unidirectional, and how they are influenced by contextual factors of disability. We also did not analyze whether differences in gender, ethnocultural background or income influenced the experience of disability. Further research is needed to determine how specific components of the framework are applicable for such groups, in other geographic settings, and to other illnesses.

## Conclusion

The *Episodic Disability Framework *provides a comprehensive overview of the dimensions, contextual factors and triggers of disability from the perspective of adults living with HIV. Features of this framework include the episodic nature of disability, a working definition of the term *episodic disability*, uncertainty as a key dimension of disablement, contextual factors that influence disability and triggers that initiate major episodes. This framework offers features beyond existing frameworks to enhance our understanding of disability experienced by adults living with HIV.

## Competing interests

The authors declare that they have no competing interests.

## Authors' contributions

KO developed the research question, study design, performed data collection and analysis and drafted the manuscript. This research was completed as part of a PhD thesis research study. AB and AD (co-supervisors) and CS and NY (committee members) participated in the development of the research question, study design, oversaw the analysis and helped to draft the manuscript. All authors read and approved the final manuscript.

## Supplementary Material

Additional file 1 **Four Dimensions of Episodic Disability**. Data describing each of the four dimensions of episodic disability: symptoms and impairments, difficulties carrying out day-to-day activities, challenges to social inclusion, and uncertainty. Click here for file
