# Supplementary material for: Exploring disability from the perspective of adults living with HIV/AIDS: Development of a conceptual framework
Source: Health Qual Life Outcomes. 2008 Oct 4;6:76. doi: 10.1186/1477-7525-6-76 (PMC2572592; doi:10.1186/1477-7525-6-76)
Supplement: Additional file 1 — Four Dimensions of Episodic Disability. Data describing each of the four dimensions of episodic disability: symptoms and impairments, difficulties carrying out day-to-day activities, challenges to social inclusion, and uncertainty. [file 1477-7525-6-76-S1.pdf]

Additional File 1: Four Dimensions of Episodic Disability

**I. Symptoms and Impairments**<br>Symptoms and impairments were experienced at the level of a body part, structure and/or function and spanned physical, mental and emotional health domains. Symptoms and impairments were primarily related to HIV, but also included manifestations from pre-existing illnesses or conditions, such as mental health or addictions.	
Fatigue, diarrhea, nausea, headaches, pain, weakness, difficulty focusing attention / forgetfulness and other adverse effects of HIV and its treatments including changes in body composition	*"The changing body structure… I have the lipodystrophy. The back of my neck is humped and my enlarged stomach. That has affected me a lot… when I'm wearing anything without a collar I have to be very aware of it…" (INT-2, FG4)*
Stress, Anxiety & Depression: Stress, anxiety and depression were specifically described as symptoms and impairments related to HIV and its treatments. Participants recited how the continual burden of HIV left them feeling "*anxious*" and "*nervous about the future*".	*"Anxiety. I think I've gotten more anxieties since I got HIV. That's impacted on my life – I get so much anxiety…I'm always thinking, when am I going to get sick, these sorts of thoughts are always in my head... Or, how long am I going to live, or am I going to die, am I going to die alone, and all of this stuff. Should I put a will together, should I do this, and all these sorts of stresses. So… I get a lot of stress, thinking about the future… I'm feeling really good now, but what about the future..." (INT-10)*
Fear: Fear stemmed from a variety of sources that included a fear of dying, becoming ill, experiencing stigma or discrimination, returning to work, taking medications, infecting others, or losing functional and financial independence.	Fearful of their impending next episode and long term survival living with HIV, participants described the *"fear that creeps in*", "*the fear of not knowing what's going to happen next"* becoming *"more frightened…worrying about the future, and worrying about my health…*"
Decreased self-esteem, shame and embarrassment: Decreased self-esteem, shame and embarrassment resulted in a lack of	*"I get a little bit of shame. I sometimes feel gross, I feel diseased. I don't want people to touch me anyway 'cause I feel gross. The biggest thing… the medication that I'm on causes body changes, and fat*

confidence, loss of pride, diminished ego, and frustration. These challenges were often linked to changes in body composition, which were sometimes attributed to antiretroviral medications.	*and stuff...” (INT-10)*
Loneliness: Being diagnosed with HIV made individuals feel “*marginalized*”, “*different*”, and “*not normal*”, which led to feelings of social exclusion, regardless of whether they showed any physical attributes of illness.	*“the moment that you test HIV-positive, you feel like someone different, you feel like a different person. And regardless of whether you’re sick or you look sick or you’re not sick… it begins already to exclude you from mainstream society.” (INT-11)*
**II. Difficulties with Day-to-Day Activities** The second dimension of *episodic disability* was difficulties carrying out day-to-day activities such difficulty walking, climbing stairs, activities of daily living, household chores, and grocery shopping. Difficulties fluctuated according to specific illness events (e.g. stroke), or fluctuated on a daily basis (e.g. in response to varying levels of energy and activity tolerance).	
**III. Challenges to Social Inclusion** The third dimension included challenges to social inclusion. These involved difficulty engaging in social roles and activities as a result of HIV disease or associated treatments.	
Parental Roles: Mothers described a conflict between the challenges of parenting and their own health. While children’s needs were often a priority over their own concerns, some health-related challenges overtook their ability to fulfill their role as a parent. Difficulty maintaining relationships with family members also was an issue. While some participants reported having supportive familial relationships, others experienced longstanding stigma from family members who were not accepting of their HIV status.	*“I don’t want to have to be promising my kids all the time, ‘Okay, we’re going to go to Wonderland this weekend,’ then we can’t go because I’m not feeling good.”…so the next day I’m on the toilet every 20 minutes. Those are the days I want to go out with the kids, and I’m thinking, oh God, my stomach’s hurting me, what if I go out, I’m going to have to be finding a bathroom all the time…They understand. Sometimes… I ask somebody else to bring them…But most of the time it’s me they want to go with.” (INT-9)*

Work & School**:** Most participants were not working, but many expressed considerations of returning to work or school. Many sought work for the opportunity to build social networks with colleagues, to enhance their financial status, and to obtain a sense of fulfillment. However, participants were cautious about whether they could handle the physical and emotional demands of the workforce given the unpredictable and variable nature of their illness. Some feared rejection by employers, colleagues, or fellow students. Others discussed fear of losing their ability to re-instate their income support program and other benefits if they experienced a serious episode of illness that left them unable to work. One participant specifically sought in full time contract work with flexible hours. She worked as much as possible while 'healthy' in order to financially anticipate or plan for episodes of illness that might leave her unable to work in the future.	*"I get too tired, and it's impacted the same way with school, I can't do a full course load, and I can't work a full day, I can only work part-time. So before I used to work full-time and then some, I was really driven to work a lot of hours. And I liked doing that. Now I can't do that. I need to take more rests. And now, I have to look for specific kinds of jobs that are, sensitive about health issues. I might need flexible work, like I might get sick or something and need time off…" (INT-9)*
Difficulty Initiating Friendships or Personal Relationships: Some participants were reluctant to engage in new relationships for fear of rejection after disclosing their HIV status. Some avoided relationships altogether to prevent feeling dishonest for not disclosing their HIV status immediately after meeting someone. Those who had experienced stigma were more selective in choosing new relationships and only disclosed their status to those who they believed were educated and would not discriminate. For some, seeking out intimate relationships was not a	*"I think my attitude towards relationships has changed, I'm more picky about who I'm friends with. Before I wasn't, and I was just friends with whoever's nice. Now I'm like, is this person educated, how much do they know, are they open-minded?" (INT-10)*

priority in the larger context of living with HIV. Their priority became taking care of their own health, and/or caring for their children, while avoiding any potential stress or anxiety that might arise from initiating a new relationship.<br><br>***Engaging in other Social Activities:*** Examples included the inability to travel to countries that prohibit visitors who are HIV-positive, sanctions against joining the military, and an inability to become more of an advocate for people living with HIV for fear of disclosure.	
**IV. Uncertainty**<br>A fourth dimension of *episodic disability* was uncertainty. Participants described how HIV "*weighed on the mind*" or "*haunted*" their lives given the unpredictable nature of the illness. Episodes were often exacerbated after receiving word of a diagnosis or experiencing a serious illness. Uncertainty also affected life decisions such as returning to work, attending school, purchasing a home, having children, or starting antiretroviral medications.	
Uncertainty & Worrying About the Future: Uncertainty was closely related to stress, anxiety and depression, and sometimes prompted feelings of fear. While participants described their understanding of death as an eventual outcome of living with HIV, the variable disease course left them wondering when their next episode might arise, the nature, severity, and consequences of that episode.	*"It haunts you in that you're not sure how long you are going to live, in that you're never sure when you're going to be sick,… you don't know how people are going to treat you if they find out, it haunts you in your place of work, in your abilities, and how it will affect your abilities in the future, or even in the present…" (INT-12)*
